# Cytotoxic Effects of *Thymus serpyllum* L. and *Mentha × piperita* L. Essential Oils on Basal Cell Carcinoma—An In Vitro Study

**DOI:** 10.3390/life15081296

**Published:** 2025-08-14

**Authors:** Maja Milosevic Markovic, Boban Anicic, Milos Lazarevic, Milica Jaksic Karisik, Dijana Mitic, Branislav Milovanovic, Stefan Ivanovic, Ilinka Pecinar, Milan Petrovic, Masa Petrovic, Nikola Markovic, Milovan Bojic, Nada Petrovic, Slobodan Petrovic, Jelena Milasin

**Affiliations:** 1 Department of Human Genetics, School of Dental Medicine, University of Belgrade, 11000 Belgrade, Serbia; 2 Clinic of Maxillofacial Surgery, School of Dental Medicine, University of Belgrade, 11000 Belgrade, Serbia; 3 Institute for Cardiovascular Diseases “Dedinje”, 11000 Belgrade, Serbia; 4 Faculty of Medicine, University of Belgrade, 11000 Belgrade, Serbia; 5 Institute for Chemistry, Technology and Metallurgy, University of Belgrade, 11000 Belgrade, Serbia; 6 Department of Agrobotany, Faculty of Agriculture, University of Belgrade, 11000 Belgrade, Serbia; 7 Development and Production Center BIOSS—Petrović IN, 11000 Belgrade, Serbia

**Keywords:** basal cell carcinoma, cancer stem cells, *Thymus serpyllum* L., *Mentha × piperita* L., essential oil

## Abstract

This study investigated the potential of *Thymus serpyllum* L. and *Mentha × piperita* L. essential oils (EOs), known for their bioactive properties, as adjunctive treatments targeting Basal cell carcinoma cancer stem cells (BCC CSCs). Primary cultures were established from ten BCC tumor samples and their distant resection margins as controls. The chemical composition of the EOs was analyzed by gas chromatography–mass spectroscopy (GC-MS) and attenuated total reflectance Fourier transform infrared spectroscopy (ATR-FTIR). The biological effects were evaluated via colony and spheroid formation, scratch assays, MTT and neutral red cytotoxicity assays, and qRT-PCR for Hh (SHH, PTCH1, SMO, and GLI1) and Notch (Notch1 and JAG1) gene expression. GC analysis identified thymol, p-cymene, and linalool as the main components of the EO of *T. serpyllum* L., and menthone and menthol in the EO of *M. × piperita* L. IC50 values were 262 µg/mL for *T. serpyllum* L. and 556 µg/mL for *M. × piperita* L. and were applied in all experiments. Both EOs significantly reduced CSC clonogenicity and migration (*p* < 0.05). The EO of *T. serpyllum* L. downregulated SMO and GLI1, while the EO of *M. × piperita* L. upregulated PTCH1, Notch1, and JAG1 (*p* < 0.05). These findings suggest that both EOs exhibit anticancer effects in BCC CSCs by modulating key oncogenic pathways, supporting their potential in BCC therapy.

## 1. Introduction

Non-melanoma skin cancers represent the most prevalent form of cancer worldwide, with an estimated global incidence rate of 79.1 cases per 100,000 individuals [1]. Among these, basal cell carcinoma (BCC) is the most common subtype [2]. Although BCC typically demonstrates slow growth and a low metastatic potential, it is nonetheless characterized by its local invasiveness, frequently resulting in substantial tissue destruction [3]. In the absence of timely diagnosis and intervention, BCC can lead to significant clinical complications and increased healthcare burdens [4,5,6]. The incidence of BCC continues to rise, particularly among younger populations, establishing it as a growing public health concern [7,8,9].

While surgical excision remains the first-line treatment for BCC, in some cases, the disease can progress to advanced or metastatic forms, necessitating targeted combination therapies [10]. This phenomenon is often attributed to the presence of cancer stem cells, which drive recurrence and therapeutic resistance through their intrinsic ability to self-renew and evade conventional treatments [11]. A key driver in BCC pathogenesis is aberrant activation of the Hedgehog (Hh) signaling pathway [12], which promotes tumor growth and sustains the proliferation of cancer stem cells in various malignancies, including prostate, pancreatic, colorectal, and breast cancers, as well as rhabdomyosarcoma and leukemia [13]. Under physiological conditions, Hh signaling is negatively regulated by Patched1 (PTCH1), which inhibits the activity of Smoothened (SMO), the key upstream activator. Upon pathway activation, SMO triggers a cascade involving the suppressor of fused homolog (SUFU), ultimately culminating in the activation of GLI transcription factors that regulate genes responsible for cell growth and proliferation [14]. In our previous study we have also stressed the importance of the PTCH1/GLI axis [15].

Although Hh pathway inhibitors have demonstrated clinical efficacy in the treatment and management of locally advanced and metastatic BCC [16,17], evidence suggests that residual tumor cells can reinitiate growth upon the cessation of treatment, thereby contributing to disease progression [18]. Additionally, the Notch signaling pathway, which is dysregulated in various hematologic malignancies and solid tumors [19,20], has been implicated in BCC development [21,22]. Notch activity is also closely associated with the tumor’s therapy responsiveness where low Notch levels confer treatment resistance, whereas high Notch activity, on the other hand, enhances apoptosis [18].

Essential oils (EOs), volatile secondary metabolites derived from aromatic plants, have been shown to exhibit not only diverse preventive and therapeutic properties but also potential anticancer properties [23,24,25,26]. Of particular interest are the EOs derived from *Thymus serpyllum* L. and *Mentha × piperita* L., which have demonstrated notable therapeutic potential [27,28,29,30,31].

*Thymus serpyllum* L. (wild thyme), a member of the *Lamiaceae* family, is an aromatic plant widely distributed across temperate regions and includes approximately 350 species worldwide [32]. Species within the *Thymus* genus are well known for their medicinal properties due to their diverse biological and pharmacological activities [33]. Various extracts of *T. serpyllum* L. are commonly used for their antiseptic, anthelmintic, carminative, expectorant, sedative, and tonic properties, as well as for their potential anticancer effects [27,28,34,35]. The cytotoxic effects of *T. serpyllum* L. extract have been previously demonstrated against lung, colon, breast, cervical, and leukemia human cancer cells [36,37,38].

*Mentha × piperita* L. (peppermint), also a member of the *Lamiaceae* family, is widely used in food, flavoring, and traditional medicine due to its diverse bioactive properties. It has gained broad application due to its antibacterial, antifungal, antiviral, and cytotoxic effects [39]. Recent studies have shown that *M. × piperita* L. extract exhibits cytotoxic activity against several tumor types, including lung, breast, and colon cancers, highlighting its potential as a promising antitumor agent [30,40,41].

Gas chromatography–mass spectrometry (GC-MS) is the most commonly used analytical methods for the characterization of the chemical composition of EOs [39]. Alternatively, some spectroscopic methods, like Fourier-transformation infrared (FT-IR) and Raman, have been successfully applied to identify the main constituents of the oil and to distinguish different species/chemotypes of different spice plants [42]. These techniques offer simple, rapid, cost-effective, and non-destructive alternatives for routine qualitative analysis.

Despite the well-documented antineoplastic properties of *T. serpyllum* L. and *M. × piperita* L. EOs, their potential as adjunctive therapies in the treatment and management of BCC remains insufficiently investigated. The aim of this study was to evaluate the anticancer effects of *T. serpyllum* L. and *M. × piperita* L. EOs on BCC cells.

## 2. Materials and Methods

### 2.1. Plant Material and Essential Oil Extraction

The *T. serpyllum* L. and *M. × piperita* L. herbs utilized in this study were obtained from Bavanište, Serbia (latitude: 44°48′59.99″; longitude: 20°52′59.99″). Voucher specimens were deposited at the Herbarium of the Institute for Medicinal Plant Research “Dr. Josif Pančić”, Belgrade (voucher No. 302,311 for *T. serpyllum* L. and voucher No 301,131 for *M. × piperita* L.). EOs were isolated from plants’ leaves via hydro-distillation using a semi-industrial SP-130 distillation unit, as previously described [43,44]. Briefly, dried plant material was isolated by a hydro-distillation process using a semi-industrial distillation unit, SP-130, which operates on the principle of water and steam distillation. During the hydro-distillation process, the temperature within the SP-130 unit ranged from 100 °C to 102 °C at atmospheric pressure.

The essential oil extracted from *Thymus serpyllum* L. was pale yellow in color, whereas the oil from *Mentha × piperita* L. exhibited a faint greenish-yellow, nearly transparent hue. Both oils had characteristic aromas consistent with their respective herbal origins. The SP-130 is a semi-industrial distillation apparatus with a total capacity of 130 L. This steam distillation method offers several advantages over traditional distillation techniques. In large-volume distillers, it is often challenging to maintain control over steam behavior and to preserve the essential properties of the oil feedstock, especially in the lower layers. In contrast, the SP-130 unit facilitates the simultaneous separation of oil and water through a condensation process, eliminating the need for additional equipment. The hydrostatic pressure during the distillation process did not exceed 101.325 KPa, ensuring optimal conditions for oil extraction.

### 2.2. Gas Chromatography–Mass Spectroscopic (GC-MS) Analysis

Gas Chromatography–Mass Spectroscopic (GC-MS) analysis was performed using an Agilent 7890A system (Agilent Technologies, Santa Clara, CA, USA) equipped with 5975C MSD and FID detectors. For chromatographic separation a weakly polar HP-5MS (5% diphenyl- and 95% dimethyl-polysiloxane, 30 m × 0.25 mm, 0.25 μm film thickness) capillary column was used (Agilent Technologies, Santa Clara, CA, USA). EO samples were dissolved in dichloromethane in the concentration of 10 µL/mL and were injected in 1:10 split mode. Helium was used as the carrier gas, at a constant pressure condition (16.255 psi). The oven temperature was programmed from 60 °C to 300 °C at a rate of 3 °C/min, and with a final hold of 10 min. Mass spectra were obtained using the electron ionization (EI) mode across a scan range of 40–600 *m*/*z*, with the ion source maintained at 230 °C, the quadrupole set to 150 °C, and a solvent delay of 3 min.

Data processing was carried out using MSD ChemStation (Agilent Technologies, Santa Clara, CA, USA). Compound identification was performed by matching the experimental mass spectra with commercial libraries (NIST 17, Wiley 7, and Adams 4) using AMDIS 32 software (v2.73) along with the NIST search program (v2.3). Retention indices (RIs) were calculated using a homologous series of *n*-alkanes (C_8_–C_32_) and compared with the literature values. Relative abundances of the detected compounds were determined from GC-FID chromatograms.

### 2.3. Attenuated Total Reflectance Fourier Transform Infrared (ATR-FTIR) Spectroscopy

ATR-FTIR analysis of *T. serpyllum* L. and *M. × piperita* L. EO samples were recorded using an IRAffinity-1 FTIR spectrometer (Schimadzu, Kyoto, Japan) system. The spectra were recorded in the spectral range from 400 to 4000 cm^−1^ with a resolution of 4 cm^−1^ (256 scans). Afterwards, preprocessing including baseline correction, normalization, and smoothing, was performed using the Spectragryph v1.2.15 software (Menges, 2018).

### 2.4. Cell Cultures

Primary basal cell carcinoma (BCC) cells and matched healthy resection margin cells (>5 mm from the tumor edge) were isolated from 10 patients, utilizing the isolation method as previously described by Milosevic et al. [45]. The study was approved by the institutional Ethics Committee of the University of Belgrade (36/30) in the Republic of Serbia and conducted in accordance with the Declaration of Helsinki.

After surgery, fresh tissue samples were cut with blades into small pieces and seeded onto T75 cell culture flasks containing Dulbecco’s Modified Eagle Medium (DMEM) supplemented with 10% fetal bovine serum (FBS) and 100 U/mL penicillin-streptomycin solution (AB) all from Thermo Fisher Scientific, Inc., Waltham, MA, USA. The cells were cultivated in a humidified atmosphere under standard conditions at 5% CO_2_ and 37 °C. The medium was discarded and changed every two to three days, and cells were passaged after reaching 80% of confluence. Contaminating fibroblasts were selectively removed using 0.125% trypsin and 0.02% edetic acid (Thermo Fisher Scientific, Inc., Waltham, MA, USA) [46]. All experiments were performed using fifth-passage tumor cells, which are enriched in cancer stem cell (CSC) populations, as shown in our prior work [45]. All experiments were conducted in triplicate.

### 2.5. MTT Cytotoxicity Assay

Primary BCC and control margin cells were seeded at a density of 1 × 10^4^ cells per well in 96-well culture plates and cultured for 24, 48, and 72 h in complete growth medium. Cells were then treated with various concentrations of the essential oils, ranging from 1 to 1000 μg/mL, dissolved in dimethyl sulfoxide (DMSO, Sigma-Aldrich, St. Louis, MO, USA) in complete growth medium [47]. Negative (no cells), solvent (0.02% DMSO), and positive controls (cells in complete growth medium plus DMSO) were also included. During this time period the medium was not refreshed. After incubation periods, cells were washed with 100 µL phosphate-buffered saline (PBS), and 100 µL of MTT solution (both from Sigma-Aldrich, St. Louis, MO, USA) was added and incubated for 3 h [48]. Following the dissolution in isopropanol, the absorbance at 560 nm was measured using a microplate reader (RT-2100c, Rayto, Shenzhen China). The cytotoxic effect of the treatment was expressed as the percentage of viability compared to the untreated cells. The toxicity of the compounds was determined by means of the formula:

Cell viability (%) = Absorbance of sample cells/Absorbance of untreated cells × 100

IC_50_ values (concentration required to reduce viability by 50%) were derived from triplicate experiments.

### 2.6. Neutral Red Assay

Cancer cells and margin cells were seeded in a 96-well plate, incubated, and treated under the same conditions as described in the previous assay. After 24 h of incubation the solutions were removed and replaced with 150 μL of Neutral red solution (3-amino-7-dimethylamino-2-methylphenazine hydrochloride; Sigma-Aldrich, St. Louis, MO, USA) and incubated for 4 h at 5% CO_2_ and 37 °C. After washing with PBS, 150 μL of Neutral red eluent (96% of ethanol: distilled water (dH_2_O): CH_3_COOH 50:49:1) solution was added to each well and incubated at room temperature for 15 min [49]. Finally, the absorbance was measured at 560 nm using a microplate reader (RT-2100c, Rayto, Shenzhen, China) and the obtained values were presented as percentages of viability.

### 2.7. Colony Forming Assay

Single-cell suspensions (200 cells/dish) from BCC and margin tissues were seeded into 35 mm Petri dishes and cultured in DMEM + 10% FBS for 24 h. Cells were then treated with the IC_50_ dose of *T. serpyllum* L. and the IC_50_ dose of *M. × piperita* L. dissolved in DMEM for the next fourteen days. Untreated control cells were cultivated in standard medium for the same period. The medium was changed every two days. The cell colonies were then washed with PBS, fixed in 4% formaldehyde for 5 min, and stained with 0.05% crystal violet for 30 min. After washing, the colonies were counted using the ImageJ software 1.48 version (NIH, Bethesda, MD, USA) (Java 1.8.9_66) and colonies containing more than 50 cells were counted as positive.

### 2.8. Spheroid Formation Assay

The CSCs and margin cells (1 × 10^4^ cells per well) were seeded in a 12-well plate coated with Poly Hem-a (Poly (2-hydroxyethyl methacrylate)). Cells were cultivated in DMEM/F12 supplemented with B27, N2, EGF, and bFGF with the addition of an IC_50_ dose of *T. serpyllum* L. or *M. × piperita* L. EO [50]. The cells were cultivated in standard medium with the aforementioned supplements as the control. After seven days of incubation, the number and diameter of the spheroids were measured using an inverted microscope (BIB-100/T, BOECO, Hamburg, Germany) and analyzed with Scope Image 9.0 software 1.48 version (NIH, Bethesda, MD, USA) (Java 1.8.9_66).

### 2.9. Scratch Wound Healing ASSAY

CSCs and margin cells (2 × 10^4^ cells per well) were plated in 24-well plates and incubated under standard conditions with DMEM. After achieving confluence of approximately 80%, the monolayer was scratched with a sterile 1.3 mm-wide rubber across the center of the well in a straight line to generate a wound in the cell monolayer and washed three times with PBS. Thereafter, the cells were treated with an IC_50_ dose of *T. serpyllum* L. and *M. × piperita* L. dissolvent in DMEM for the next 72 h. For the control, the cells were also cultivated in standard medium for the same period. Photographs were made at 72 h after the treatment using an inverted microscope (BIB-100/T, BOECO, Hamburg, Germany) and HDCE-90D camera (BOECO, Hamburg, Germany). Wound areas were analyzed with Scope Image 9.0 software 1.48 version (NIH, Bethesda, MD, USA) (Java 1.8.9_66) to measure the closest area of the scratch and the migration speed was calculated independently at 24 h intervals (e.g., 0 h, 24 h, 48 h, and 72 h) [51].

### 2.10. RNA Extraction

Total RNA was extracted using TRIzol Reagent (Invitrogen, Thermo Fisher Scientific, Waltham, MA, USA). First-strand cDNA synthesis was performed with 2 µg of total RNA using Oligo (dT) primers (Invitrogen, Thermo Fisher Scientific, Waltham, MA, USA) and RevertAid reverse transcriptase (Thermo Fisher Scientific, Waltham, MA, USA). RNA was isolated from margin cells, untreated CSCs, and CSCs treated with *T. serpyllum* L. and *M. × piperita* L. EO and DMSO.

### 2.11. Gene Expression Analysis of Signaling Markers

For quantitative real-time PCR (qPCR) analysis, cDNA was amplified by Taq DNA polymerase. Subsequent qPCR was performed using the Line Gene-K Fluorescence Real-time PCR Detection System (Bioer, Binjiang, Hangzhou, China) using the Maxima™ SYBR Green/ROX qPCR Master Mix (Thermo Fisher Scientific, Waltham, MA, USA). The expression of markers of Sonic Hedgehog (SHH) signaling cascade (SHH, PTCH1, SMO, and GLI1) and Notch signaling pathway (Notch 1 and JAG1) were analyzed under the same conditions. The housekeeping gene GAPDH was used as reference. Fold-induction values were calculated using the 2^−ΔCt^ method. The sequences of all primers used in the experiments are given in Table 1.

#### Statistical Analysis

Statistical analyses were conducted using SPSS software version 22.0 (SPSS Inc., Chicago, IL, USA). Student’s *t*-test was used to determine statistical significance, with a *p*-value < 0.05 considered significant.

## 3. Results

### 3.1. Gas Chromatography–Mass Spectroscopic (GC-MS) Analysis

**Figure 1:**
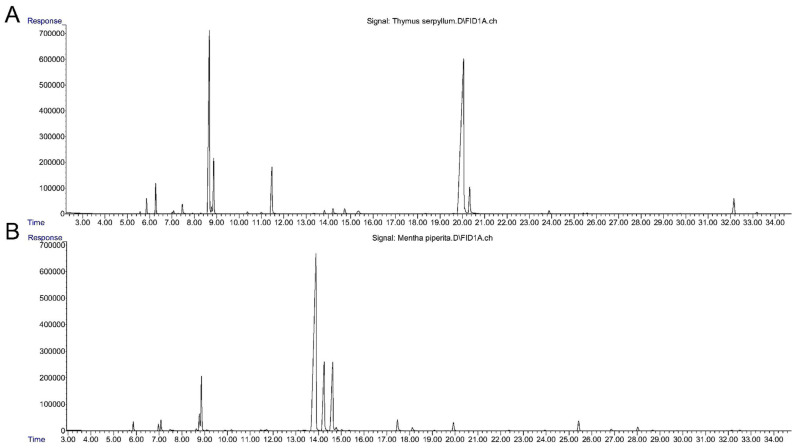
GC-FID chromatograms of (**A**) *T. serpyllum* L. and (**B**) *M. × piperita* L. essential oils. In *T. serpyllum* EO, the predominant compound was thymol (50.47%), followed by p-cymene (23.56%), linalool (5.84%), 1,8-cineole (4.98%), carvacrol (3.18%), camphene (2.33%), and caryophyllene oxide (2.28%). In *M. × piperita* EO, the main constituents were menthone (53.66%), menthol (13.52%), iso-menthone (6.64%), menthofuran (6.53%), and 1,8-cineole (6.13%).

GC-FID-MS analysis of the essential oils (EOs) from *Thymus serpyllum* L. and *Mentha × piperita* L. revealed distinct chemical profiles, primarily composed of monoterpenes. A total of 36 compounds were identified in *T. serpyllum* L. EO and 53 in *M. × piperita* L. EO. Comprehensive chemical compositions are detailed in Table 2 and Table 3, and corresponding GC-FID chromatograms are shown in Figure 1.

### 3.2. Attenuated Total Reflectance Fourier Transform Infrared Spectroscopy (ATR-FTIR)

**Figure 2:**
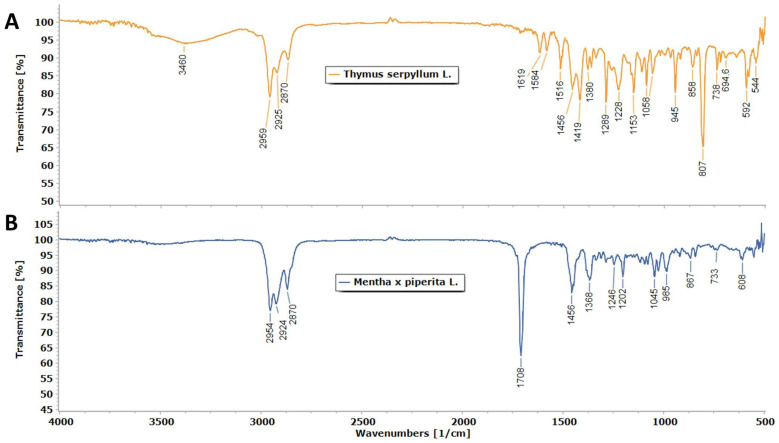
Average FT-IR spectrum of (**A**) *T. serpyllum* L. and (**B**) *M. × piperita* L. essential oils.

ATR FT-IR spectra and the main vibrational bands of the EOs in the spectral range from 500 to 4000 cm^−1^ are shown in Figure 2A and 2B, respectively.

The bands with higher intensity indicate the dominant component, which is indicative of the content of monoterpenes and their metabolic precursors [52]. The prominent peaks of the *Thymus serpyllum* essential oil sample were observed at 2959, 2925, 2870 (C–H stretching in aliphatic groups), 1619, 1584, and 1456 (C=C st), 1419, 1380 (CH_2_ δ and CH_3_ δ), 1289, 1228, 1088, 945 (C–O–H st), 807, 592 (C–H wagging vibrations), and 738 (CH_2_ γ) cm^−1^ as shown in Figure 2A. According to the literature these bands indicate monoterpene hydrocarbons, oxygenated monoterpenes, and sesquiterpene hydrocarbons [53]. The prominent bands for the *Mentha × piperita* essential oil sample were observed at 2954, 2924, 2870 (C–H stretching of CH_3_ and CH_2_ groups), 1708 (C=O stretching vibrations), 1456, 1366 (CH2 δ and CH3 δ), 1246, 1202 (C–O st), 1045, 985 (–HC=CH–), 967, 733 (CH_2_ γ), and 608 cm^−1^ as shown in Figure 2B. According to the literature, these bands primarily indicate the alcohol group and the ketone group from monoterpenes [54].

### 3.3. MTT Assay

**Figure 3:**
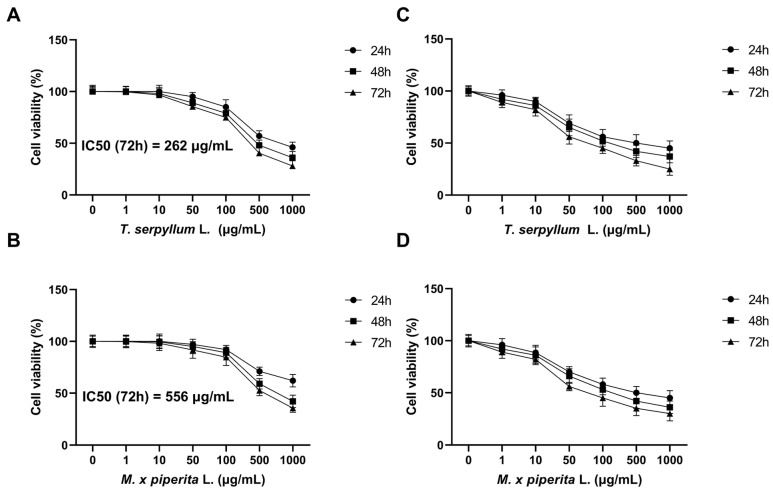
Dose- and time-dependent inhibition of BCC stem cells viability exerted by essential oils of *T. serpyllum* L. (**A**) and *M. × piperita* L. (**B**). Cytotoxicity was determined by the MTT (**A**,**B**) and Neutral Red (**C**,**D**) assays. The results are expressed as the mean of triplicate (±SD).

To assess the cytotoxic potential of the EOs, BCC cancer stem cells (CSCs), and control margin cells were treated with increasing concentrations of *T. serpyllum* and *M. × piperita* EOs (1–1000 µg/mL) for 24, 48, and 72 h. MTT assay results demonstrated a clear time- and dose-dependent decrease in BCC CSC viability. As the concentration of essential oils increased (1–1000 µg/mL), cell viability progressively declined at all time points (24, 48, and 72 h). The most pronounced effect was observed after 72 h of treatment, with *T. serpyllum* reducing viability to approximately 28% and *M. × piperita* to about 35% at the highest dose (Figure 3A,B). After 72 h, the IC_50_ values were calculated to be 262 µg/mL for *T. serpyllum* and 556 µg/mL for *M. × piperita* (Figure 3). In contrast, no statistically significant reduction in cell viability was observed in healthy margin cells under the same conditions.

### 3.4. Neutral Red (NR) Assay

To validate the MTT results, a complementary Neutral Red (NR) assay was conducted. The NR assay confirmed the MTT findings, demonstrating a comparable dose- and time-dependent decrease in the viability of BCC CSCs. As observed with the MTT assay, cell viability declined progressively with increasing essential oil concentrations and longer exposure durations. The most pronounced reduction was seen after 72 h at the highest concentration (1000 µg/mL), with viability dropping to 25% for *T. serpyllum* and 30% for *M. × piperita* (Figure 3). No statistically significant reduction in the viability of healthy margin cells was observed in the NR assay.

### 3.5. Colony Forming Assay

**Figure 4:**
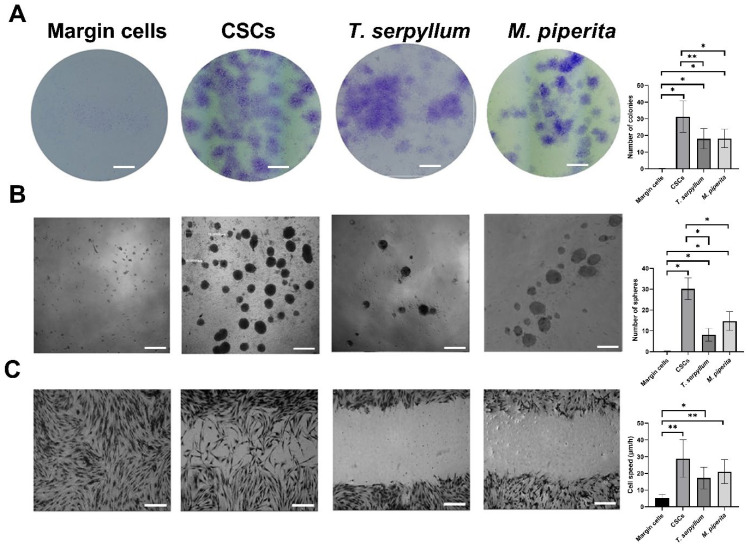
Colony forming, Spheroid formation, and Scratch wound healing assay with quantitative analysis (representative images at higher magnification 100×, scale bar: 200 µm). Treated and untreated CSCs showed higher clonogenic ability (**A**) and the ability to form tumorsphere (**B**) compared to margin cells. There was a significantly decreased capacity of tumor cells to form a colony (**A**) and spheres (**B**) after treatment with *T. serpyllum* L. and *M. × piperita* L. Representative images of migratory potential. There is a statistically significant difference in cell speed between tumor cells (CSCs and CSCs treated with *T. serpyllum* L. and *M. × piperita* L.) and margin cells (**C**). In the quantitative analysis, error bars represent standard deviation calculated from experiments, while asterisks * and ** designate *p*-values lower than 0.05 and 0.01, respectively.

Clonogenic potential was evaluated by a colony forming assay. After fourteen days, BCC CSCs formed colonies unlike control margin cells (*p* < 0.05). The potential of forming colonies was significantly reduced after BCC CSC treatment with *T. serpyllum* L. and *M. × piperita* L. (*p* < 0.05) compared to untreated CSCs (Figure 4A).

### 3.6. Spheroid Formation Assay

After seven days, both treated and untreated BCC CSCs formed spheroids unlike healthy margin control cells (Figure 4B) (*p* < 0.05). The number of spheres was significantly smaller after treatment with *T. serpyllum* L. and *M. × piperita* L. compared to untreated cells (*p* < 0.05). The number of tumor spheres was similar, while the diameter after treatment with *T. serpyllum* L. was smaller (117 ± 21) compared to treatment with *M. × piperita* L. (148 ± 23 µm).

### 3.7. Scratch Wound Healing Assay

After the formation of a scratch in the cell layer, the distance between the cells was measured every 24 h and the average velocity of the cells was calculated (Figure 4C). The space between the cells was filled after 72 h and there was a statistically significant difference in speed between CSCs and healthy margin control (*p* < 0.05). There was a decrease in cell speed after treatment with extracts of both essential oils but without a statistically significant difference.

### 3.8. Gene Expression Analysis: Sonic Hedgehog and Notch Signaling Pathway

**Figure 5:**
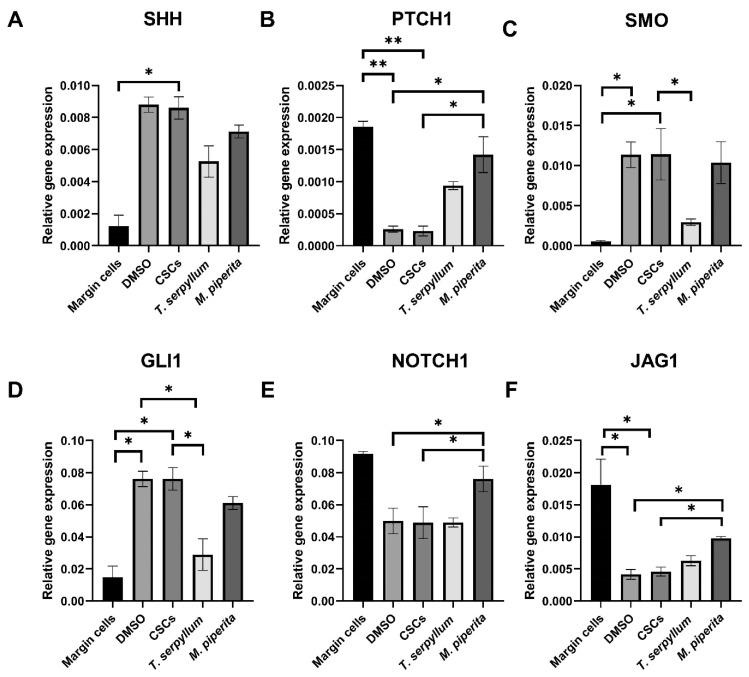
The gene expression analysis of the Sonic Hedgehog and Notch signaling pathway in BCC CSCs after treatment with *T. serpyllum* L. and *M. × piperita* L. The mRNA levels of Sonic Hedgehog markers SHH, SMO, and GLI1 were significantly higher in tumor cells compared to the healthy control (**A**,**C**,**D**). There was a significant decrease in SMO and GLI1 levels after treatment with *T. serpyllum* L. compared to untreated cells (**C**,**D**). PTCH1 was significantly lower in tumor cells compared to the healthy control and there was a significant increase in the expression of this gene after the treatment with *M. × piperita* L. (**B**). The level of Notch 1 and JAG1 genes was lower in CSCs compared to margin cells (**E**,**F**). After treatment with *M. × piperita* L. there was a significant increase in the expression of these genes, while after treatment with T. serpyllum L., there was no difference (**E**,**F**). The error bars represent the standard error calculated from experiments. Asterisks * and ** designate *p*-values lower than 0.05 and 0.01, respectively. Abbreviation: SHH—Sonic Hedgehog Signaling Molecule; PTCH1—Patched 1 Receptor; SMO—Smoothened, Frizzled Class G protein-coupled Receptor; GLI1—glioma-associated oncogene 1.

The expression of Hedgehog pathway genes (SHH, SMO, and GLI1) was significantly higher in BCC CSCs than in margin cells (healthy control) (*p* < 0.05) (Figure 5A,C,D). Treatment with *T. serpyllum* significantly reduced SMO and GLI1 levels as compared to untreated CSCs (*p* < 0.05) (Figure 5C,D), while SHH was downregulated without reaching statistical significance (Figure 5A). PT TCH1 was expressed at lower levels in CSCs than in margin cells but was significantly upregulated following *M. × piperita* L. treatment (*p* < 0.05) (Figure 5B).

Regarding the Notch signaling pathway, Notch1 and JAG1 expression levels were significantly lower in CSCs compared to margin cells (*p* < 0.05) (Figure 5E,F). Following treatment with *M. × piperita* L., a significant overexpression of both genes (*p* < 0.05) was observed, whereas *T. serpyllum* L. had no significant effect.

## 4. Discussion

Over the past decade, considerable research has been directed toward the development of novel therapeutic agents aimed at minimizing the adverse effects associated with conventional treatments. In this context, there is a growing interest in bioactive compounds derived from plants, which offer the potential for high efficacy, low toxicity, and minimal environmental impact [55]. The use of plant extracts for the prevention and treatment of various pathological conditions, including cancer, has attracted growing scientific interest. The antitumor properties of *T. serpyllum* and *M. piperita* EOs have been previously reported [27,28,29,30]. In the present study, we demonstrated that these EOs also exert antitumor activity against BCC CSCs.

In our study, GC-MS analysis revealed that thymol and *p*-cymene are the major constituents of the *T. serpyllum* L. EO, which is in agreement with previous reports [36,56,57,58,59]. The literature data suggest that thymol, the principal bioactive component of thyme essential oil, exhibits anticancer potential, in addition to a broad range of pharmacological activities [36,60]. In our study, thymol was identified as the most dominant component in *T. serpyllum* L. EO (50.47%), which is consistent with the spectroscopic findings reported in other studies [59,61]. Regarding *p*-cymene, researchers from geographically close regions, such as Montenegro, have reported similar relative percentages to ours [37].

The present study determined that the relative percentages of linalool (5.84%) and 1,8-cineole (4.98%), were slightly higher than previously reported for the same geographic region [62]. The variability in the concentration of aromatic compounds in *T. serpyllum* L. reported by different studies is generally attributed to factors such as time of harvest, soil characteristics, the extraction techniques employed, etc. [37,63].

The main components of the *M. × piperita* L. EO are menthone and menthol, followed by *iso*-menthone, menthofuran, and 1,8-cineole, which aligns with the findings from other studies [36,64,65]. Many sources state that menthol can reach up to 70% of *M. × piperita* L. EO content [40,66,67]. In a wild peppermint EO from Serbia, menthol was reported as the dominant component (48.6%) [68]. However, in samples of noncommercial genuine peppermint essential oil, it was reported that the main component is menthone. Nilo et al. reported that genuine peppermint EO contains 31.43% menthone and 14.08% menthol [69]. In any case, there is growing evidence that peppermint oil and its components, in particular menthol, possess anticancer activity. Studies have shown that menthol can induce apoptosis, cause cell cycle arrest, and inhibit proliferation in various cancer cell lines [70,71].

The average ATR-FT-IR spectrum of the *T. serpyllum* L. EO shows the typical signals of the terpene functional groups and confirms the GC-MS results. The higher intensity bands at 2959, 2925, and 2870 cm^−1^ are assigned to asymmetric and symmetric C–H stretching in aliphatic –CH_3_ groups [52]. The lower intensity bands at 1584 and 1456 cm^−1^ point to the signals of C=C stretching vibration in the aromatic ring of cymene and thymol and carvacrol, respectively. Asymmetric C-H bending vibrations cause the signals at 1516 and 1380 cm^−1^, which are related to symmetric bending and stretching vibrations of methyl groups in p-cymene. The most characteristic band of carvacrol is at 858 cm^−1^, while the presence of an aromatic ring is confirmed by the typical signal at 1228 cm^−1^ such as the band related to the aromatic C–H in-plane [52]. The presence of thymol is confirmed by the absorption bands of C–O-H stretching at 1289, 1153, 1088, and 945 cm^−1^ [42]. The FT-IR spectrum of thyme EO shows an intense band at 807 cm^−1^ attributed to out-of-plane CH wagging vibrations resulting from the overlap of the thymol and p-cymene bands (804 and 813 cm^−1^, respectively) [72]. The band at 807 cm^−1^ is the most important signal for distinguishing the different types of aromatic ring substitutions. The presence of intense and sharp bands at 807, 945, 1088, 1153, and 1289 cm^−1^ allows the determination of thymol as the main constituent [42].

The FT-IR spectrum of peppermint essential oil shows bands at 2954, 2924, and 2870 cm^−1^, corresponding to symmetrical and asymmetrical C−H stretching vibrations of the aliphatic CH_3_ and CH_2_ groups in alkanes [54,73]. The strongest intensity band at 1708 cm^−1^ belongs to C=O stretching vibrations from the carbonyl group of menthone, present in the essential oil [74,75,76]. The vibrational band at 1456 and 1366 cm^−1^ could be attributed to the bending vibrations of the CH_2_ and CH_3_ groups of menthone and menthol, as the most prominent in peppermint essential oil [54,74,75,76,77]. The spectral band at 1246, 1202, and 1045 cm^−1^ can be assigned to the C‒O stretching of alcohols [73,74,78].

Our results demonstrated that *T. serpyllum* L. and *M. × piperita* L. EOs reduced the viability of BCC CSCs in a concentration-dependent manner. These findings are consistent with previous studies reporting the dose-dependent cytotoxic effects of the *T. serpyllum* L. EO on various cancer cell types, including breast, lung, colorectal, hepatocellular, and cervical cancer cells [36,37,47]. Similarly, various extracts and the essential oil of *M. × piperita* L. have shown dose-dependent cytotoxic and antiproliferative effects across multiple cancer cell lines [79,80,81,82,83].

In addition to reducing cell viability, *T. serpyllum* L. and *M. × piperita* L. essential oils were found to inhibit the clonogenic potential, sphere-forming ability, and migratory capacity of BCC cancer cells. These findings are in line with previous studies that have reported similar antiproliferative effects of the *T. serpyllum* L. essential oil on various carcinoma cell lines, including liver, colon, breast, prostate, and lung cancer cells [47,56]. Iron oxide nanoparticles synthesized using *M. × piperita* L. extract have demonstrated significant antimigratory potential against highly metastatic human breast cancer cells [84].

In cancer, the major mechanism of chemotherapeutic action is the induction of cancer cells’ death, but this effect does not bypass the normal cells. Therefore, there is a need for antineoplastic agents that will effectively destroy tumor cells with minimal effects on normal cells. Our data demonstrated that *T. serpyllum* L. does not affect healthy margin cells, a phenomenon that was also observed in normal human breast cells [47].

The Hh signaling pathway plays a central role in the development of BCC making it a promising target for therapeutic intervention. Some Hh inhibitors have been approved by the US Food and Drug Administration (FDA), such as vismodegib which is highly effective in the treatment of BCC [16]. However, it was noted that after treatment cessation, the primary tumor usually regenerates because residual tumor cells persist [85]. The reason is the existence of molecularly and functionally specific compartments in peripheral basal BCC cells which are Hh positive and Notch negative and survive the treatment [18]. This fact points to the need for adjunctive therapy that would modulate both pathways simultaneously. In the present study, we demonstrated the impact of *T. serpyllum* L. and *M. × piperita* L. EOs on the Hh and Notch signaling pathways in BCC, highlighting their dual role organized into two molecularly and functionally distinct compartments.

In BCC, the excessive activation of the Hh pathway leads to uncontrolled tumor cell proliferation. Most BCC cases arise from loss-of-function mutations in PTCH1, while a smaller proportion are driven by gain-of-function mutations in SMO [86,87]. Our results show that PTCH1 expression is suppressed in BCC CSCs compared to the margin tissue and that the treatment with both essential oils lead to an increase in its expression, with the effect of *M. × piperita* L. essential oil being statistically significant. Conversely, our findings revealed that *SMO* was markedly upregulated in BCC CSCs relative to margin cells, while treatment with *T. serpyllum* L. essential oil led to a significant downregulation of its expression.

A dysregulated Hh/GLI pathway plays a central role in BCC tumorigenesis and aggressiveness, making it a crucial therapeutic target [14,88,89]. Indeed, in our study, GLI1 was highly overexpressed in BCC cancer stem cells compared to healthy margin cells, and its expression was reduced following the treatment with both essential oils, although only *T. serpyllum* showed a statistically significant decrease.

The Notch signaling pathway, which is downregulated in basal cell carcinoma, has been implicated in promoting apoptosis upon reactivation, underscoring its potential role as a therapeutic target in managing this malignancy [90]. The *Notch1* gene encodes one of the Notch receptors, while *JAG1* encodes *Jagged1*, a single-pass transmembrane protein and one of the five Notch ligands [91]. The *Notch1* receptor plays a crucial role in cellular processes such as signaling, proliferation, differentiation, and apoptosis [19,20]. Our results clearly demonstrate that the treatment with the *M. × piperita* essential oil leads to an increased expression of Notch 1 and JAG1 in BCC cells. This is consistent with the assertion that low *Notch* levels protect tumors against treatment, while high *Notch* activity promotes cell death [18]. This could be explained by the stimulation of Notch signaling via JAG1, which induces apoptosis in BCC cells through the upregulation of Fas ligand expression and subsequent activation of the downstream caspase-8 [90].

The *T. serpyllum* L. EO has demonstrated inhibitory effects on oral cancer cells via the modulation of multiple tumor-suppressive signaling pathways, including interferon signaling, N-glycan biosynthesis, and extracellular signal-regulated kinase 5 (ERK5) pathways [92]. Furthermore, thymol isolated from the EO of the *Lamiaceae* family has been shown to inhibit colorectal cancer cell growth and metastasis by suppressing the Wnt/β-catenin pathway [93]. Although the specific effects of *T. serpyllum* L. on the Hh and Notch pathways have not yet been thoroughly investigated, the existing evidence suggests its potential relevance in managing skin malignancies such as melanoma, in which these pathways are known to play pivotal roles [94,95,96].

*Mentha × piperita* L. extract has likewise demonstrated a range of chemopreventive effects. Notably, it can inhibit the development of skin papillomas by modulating the activation and detoxification of carcinogens and enhancing resistance to radiation-induced damage [97]. The ability of *M. × piperita* to protect against UVB-induced DNA mutations—particularly those affecting RAS, TP53, and PTCH1, which are frequently implicated in BCC pathogenesis—is of particular significance [98,99]. Another study indicated that chloroform and ethyl acetate extracts of *M. × piperita* L. exerted notable anticancer effects in a dose- and time-dependent manner. These effects were associated with G1 phase cell cycle arrest, mitochondrial pathway–mediated apoptosis, disruption of redox homeostasis, upregulation of Bax, increased expression of p53 and p21, as well as the activation of pro-inflammatory cytokine responses [83]. It has also been reported that *M. × piperita* L. leaf extract, which contains rosmarinic acid and luteolin-7-O-glucuronide, has the potential to reduce extracellular ATP (eATP) release from epidermal keratinocytes—both during inflammation and as a consequence of natural aging [100].

Taken together, our findings provide compelling evidence that *T. serpyllum* L. and *M. × piperita* L. essential oils exert multifaceted antitumor effects against BCC CSCs through the modulation of key signaling pathways involved in tumorigenesis, including Hh and Notch. The observed inhibition of proliferation, migration, clonogenicity, and sphere-forming capacity, alongside selective cytotoxicity toward malignant cells and the sparing of healthy tissue, highlight their therapeutic potential. In other words, these essential oils may be a valuable adjunct to conventional treatment modalities. However, a limitation of this study is the lack of a separate assessment of the major individual compounds present in the essential oils (e.g., thymol, menthol, and menthone), which could have offered a more precise insight into the specific active components responsible for the observed effects. Future research should focus on elucidating Eos’ molecular mechanisms of action, optimizing delivery methods, and validating efficacy in in vivo models. In addition, their potential synergistic interactions should also be evaluated. These steps would ultimately pave the way for their integration into targeted strategies for BCC treatment.

## 5. Conclusions

This study highlights the potential of *T. serpyllum* L. and *M. × piperita* L. essential oils as promising sources of bioactive compounds with selective cytotoxic and anti-proliferative effects on BCC CSCs. Notably, *T. serpyllum* L. EO downregulates *SMO* and *GLI1* in the Hh signaling pathway, while *M. × piperita* L. EO upregulates *PTCH1* in the Hh pathway and *Notch1* and *JAG1* in the Notch signaling cascade. These findings underscore the ability of the two essential oils to modulate key molecular signaling implicated in BCC pathogenesis, supporting their potential as candidates for the development of novel treatment strategies.

## Figures and Tables

**Table 1:** Primers with corresponding sequences used in the study.

Product Name		Sequences (5′→3′)
SHH	Forward	GAAAGCAGAGAACTCGGTGG
	Reverse	GGAAAGTGAGGAAGTCGCTG
PTCH1	Forward	GGGTGGCACAGTCAAGAACAG
	Reverse	TACCCCTTGAAGTGCTCGTACA
SMO	Forward	GGGAGGCTACTTCCTCATCC
	Reverse	GGCAGCTGAAGGTAATGAGC
GLI1	Forward	GAAGACCTCTCCAGCTTGGA
	Reverse	GGCTGACAGTATAGGCAGAG
Notch 1	Forward	AGCCTCAACATCCCCTACAA
	Reverse	CCACGAAGAACAGAAGCACA
JAG1	Forward	CGGGATTTGGTTAATGGTTATC
	Reverse	ATAGTCACTGGCACGGTTGTAGCAC
GAPDH	Forward	TCATGACCACAGTCCATGCCATCA
	Reverse	CCCTGTTGCTGTAGCCAAATTCGT

**Table 2:** Chemical composition of *T. serpyllum* L. essential oil.

No.	Compound	RI	R.T. (min)	Relative Percentage (%)
1	Tricyclene	921	5.569	0.15
2	*α*-Pinene	931	5.859	1.13
3	*β*-Fenchene	935	5.978	0.02
4	*α*-Fenchene	944	6.228	0.04
**5**	**Camphene**	**945**	**6.269**	**2.33**
6	*trans*-*p*-Menthane	972	7.007	0.13
7	*β*-Pinene	974	7.074	0.27
8	Myrcene	988	7.459	0.94
9	*p*-Mentha-1(7),8-diene	1003	7.917	0.06
10	1,4-Cineole	1013	8.283	0.07
**11**	***p*-Cymene**	**1023**	**8.670**	**23.56**
12	Limonene	1026	8.776	0.62
**13**	**1,8-Cineole**	**1029**	**8.866**	**4.98**
14	*γ*-Terpinene	1057	9.885	0.02
15	*cis*-Linalool oxide (furanoid)	1069	10.37	0.21
16	*trans*-Linalool oxide (furanoid)	1086	10.996	0.20
**17**	**Linalool**	**1099**	**11.469**	**5.84**
18	*endo*-Fenchol	1112	12.009	0.01
19	*cis*-*β*-Terpineol	1143	13.315	0.06
20	Isoborneol	1153	13.821	0.39
21	Borneol	1162	14.206	0.66
22	Terpinen-4-ol	1174	14.727	0.73
23	*α*-Terpineol	1189	15.334	0.61
24	Linalyl acetate	1257	18.336	0.07
**25**	**Thymol**	**1295**	**20.056**	**50.47**
**26**	**Carvacrol**	**1301**	**20.327**	**3.18**
27	Neryl acetate	1365	23.058	0.02
28	*α*-Copaene	1375	23.542	0.06
29	Geranyl acetate	1383	23.876	0.43
30	Longifolene	1405	24.807	0.01
31	(*E*)-Caryophyllene	1419	25.414	0.08
32	*α*-Humulene	1454	26.858	0.01
33	*δ*-Cadinene	1524	29.751	0.05
**34**	**Caryophyllene oxide**	**1582**	**32.158**	**2.28**
35	Humulene oxide II	1608	33.186	0.23
36	14-hydroxy-(*Z*)-Caryophyllene	1670	35.575	0.07

**Table 3:** Chemical composition of *M. × piperita* L. essential oil.

No.	Compound	RI	R.T. (min)	Relative Percentage (%)
1	*α*-Thujene	926	5.688	0.02
2	*α*-Pinene	931	5.864	0.80
3	Camphene	946	6.283	0.02
4	Thuja-2,4(10)-diene	951	6.431	0.02
5	Sabinene	970	6.965	0.65
6	*β*-Pinene	974	7.078	1.11
7	Myrcene	989	7.476	0.16
8	*α*-Phellandrene	997	7.600	0.02
9	*α*-Terpinene	1016	8.380	0.02
10	*p*-Cymene	1022	8.627	0.23
11	Limonene	1026	8.764	1.91
**12**	**1,8-Cineole**	**1028**	**8.856**	**6.13**
13	(*Z*)-beta-Ocimene	1034	9.080	0.12
14	*γ*-Terpinene	1056	9.879	0.06
15	*cis*-Sabinene hydrate	1064	10.171	0.12
16	Terpinolene	1088	11.055	0.02
17	Linalool	1099	11.464	0.12
18	2-Methylbutyl 2-methylbutanoate	1103	11.628	0.05
19	*cis*-Thujone	1105	11.708	0.14
20	*trans*-Thujone	1115	12.186	0.03
21	*trans*-Sabinol	1139	13.109	0.04
22	Camphor	1142	13.330	0.06
23	*neo*-Isopulegol	1144	13.397	0.09
**24**	**Menthone**	**1155**	**13.890**	**53.66**
**25**	**Menthofuran**	**1162**	**14.194**	**6.53**
**26**	***iso*-Menthone**	**1163**	**14.245**	**6.64**
**27**	**Menthol**	**1172**	**14.623**	**13.52**
28	Terpinen-4-ol	1176	14.777	0.58
29	*iso*-Menthol	1181	15.018	0.14
30	*α*-Terpineol	1189	15.343	0.05
31	Myrtenal	1195	15.595	0.02
32	Pulegone	1237	17.467	1.68
33	Piperitone	1252	18.118	0.54
34	*neo*-Menthyl acetate	1273	19.086	0.08
35	Bornyl acetate	1284	19.559	0.03
36	Dihydroedulan	1287	19.673	0.04
37	Menthyl acetate	1292	19.927	1.27
38	*iso*-Menthyl acetate	1307	20.568	0.05
39	Menthofurolactone 1	1346	22.258	0.06
40	Menthofurolactone 2	1348	22.367	0.07
41	*β*-Bourbonene	1385	23.938	0.09
42	*β*-Elemene	1392	24.265	0.04
43	(*E*)-Caryophyllene	1419	25.422	1.58
44	*α*-Humulene	1454	26.86	0.26
45	(*E*)-*β*-Farnesene	1458	27.020	0.03
46	Germacrene D	1482	28.020	0.61
47	Bicyclogermacrene	1497	28.668	0.12
48	*δ*-Cadinene	1524	29.766	0.05
49	Spathulenol	1577	31.933	0.02
50	Caryophyllene oxide	1582	32.142	0.12
51	Viridiflorol	1591	32.500	0.12
52	6-methoxy-Elemicin	1598	32.773	0.02
53	Humulene epoxide II	1608	33.200	0.03

## Data Availability

The data presented in this study are available on request from the corresponding author.
